# Anaplastic thyroid carcinoma with chondrosarcomatous differentiation: a case report

**DOI:** 10.1186/s13000-020-01005-y

**Published:** 2020-07-21

**Authors:** Jixuan Liu, Ni Cui, Wenjia Ding, Xinjie Dong, Xiaoshuai Chen, Jun Jiang, Yafang Liu

**Affiliations:** 1 Department of Pathology, The First Hospital of Jilin University, Changchun, 130021 Jilin China; 2 Department of General Surgery, China-Japan Union Hospital of Jilin University, Changchun, 130021 Jilin China; 3 Department of Radiology, The First Hospital of Jilin University, Changchun, 130021 Jilin China

**Keywords:** Anaplastic carcinoma, Diagnosis, Differential diagnosis, Dedifferentiated chondrosarcoma, Thyroid, Case report

## Abstract

**Background:**

Anaplastic thyroid carcinoma (ATC) is a rare malignant tumor. In addition to the main ATC type with classical histopathological features, the other morphological types of ATC include paucicellular variant, angiomatoid, lymphoepithelioma-like, and small-cell variant. However, an ATC variant with a chondrosarcomatous component has not been reported to date.

**Case presentation:**

Computed tomography imaging of a 63-year-old male with a 2-month history of a cervical mass revealed a 4.5-cm lesion with heterogeneous enhancement in the left thyroid lobe and two smooth and homogeneous nodules in the right thyroid lobe. The patient underwent total thyroidectomy and cervical lymph node resection. Histologically, the tumor boundary in the left lobe was clear, with a few mitotically active, spindle sarcoma-like tumor cells observed in some areas. Immunohistochemically, these spindle cells were positive for vimentin and negative for cytokeratin, paired box-8, epithelial membrane antigen, calcitonin, thyroglobulin, and thyroid transcription factor-1. In other areas, abundant cartilage matrix production and irregularly shaped lobules of cartilage, often separated by fibrous bands, were observed. The chondrocytes appeared mildly/moderately atypical and contained enlarged, hyperchromatic nucleoli. One of the two nodules in the right thyroid lobe had a clear boundary and comprised some bland spindle cells in a prominently collagenous stroma with clear boundaries. The other nodule in the right thyroid lobe was completely enclosed within a thin, fibrous capsule and exhibited normofollicular and microfollicular architecture. The patient received adjuvant radiotherapy after the surgery and was free of any local or regional recurrence or distant metastases at the 8-month follow-up evaluation.

**Conclusions:**

This unusual case of ATC with chondrosarcomatous differentiation is an important addition to the morphology spectrum of ATC types.

## Background

Anaplastic thyroid carcinoma (ATC) is the rarest type of thyroid carcinoma, accounting for approximately 0.85% of all thyroid carcinoma cases [1]. Some of the rare variants of ATC that have been described are paucicellular variant [2], angiomatoid [3], lymphoepithelioma-like, and small-cell variant. However, ATC with a chondrosarcomatous component is extremely rare [4]. Conversely, thyroid chondrosarcoma is also extremely rare, with only few cases of primary and secondary thyroid chondrosarcomas reported in the literature [5–8]. Dedifferentiated chondrosarcoma mainly comprises cartilaginous and dedifferentiated tumor components; the differentiated component often comprises spindle sarcoma. Importantly, ATC frequently contains malignant spindle cells resembling high-grade pleomorphic sarcoma. Therefore, there is an overlap of pathological manifestations between these two rare thyroid tumors. The diagnosis can be more challenging in cases where ATC does not exhibit a typical morphology, especially when the malignant spindle cells do not express cytokeratin. We herein report an unusual case of ATC containing a chondrosarcomatous component.

## Case presentation

**Fig. 1:**
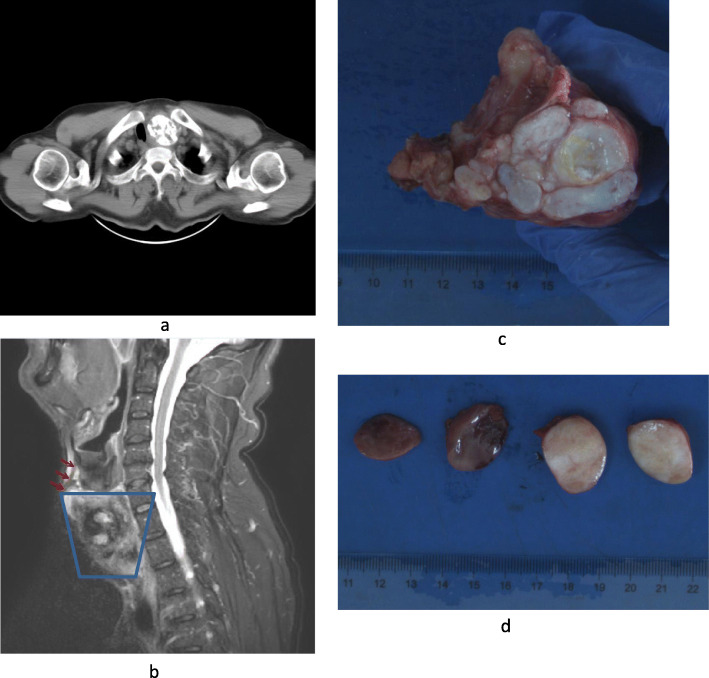
**a**. Computed tomography image showing a mass, 4.5 × 4.0 × 4.5 cm in size, with calcification in the left thyroid lobe and two smooth and homogeneous nodules in the right thyroid lobe. The trachea is compressed and deflected toward the right side. **b**. Neck MRI showed that the epicenter of the left lobe mass was in the thyroid gland and it did not originate from the surrounding thyroid cartilage (the red arrow shows the thyroid cartilage, and the blue box shows the main body of the tumor). **c**. Gross examination showed that a gray, lobulated mass occupied the majority of the left thyroid lobe. The cut surface appeared gray with hard consistency. **d**. Two homogeneous, oval nodules in the right thyroid lobe appeared gray with hard consistency

A 63-year-old male presented to the First Hospital of Jilin University with a 2-month history of a cervical mass. He had no history of neoplasms and no family history of tumors. Blood tests showed slightly increased thyroid stimulating hormone levels (56 μIU/mL) and decreased T3 (2.4 pmol/L) and T4 (10 pmol/L) levels. His antithyroglobulin antibody levels were within the normal range. Computed tomography imaging showed a mass, approximately 4.5 × 4.0 × 4.5 cm in size, with calcification in the left thyroid lobe and two smooth and homogeneous nodules in the right thyroid lobe. The trachea was compressed and deflected toward the left side (Fig. 1a). The patient underwent total thyroidectomy with cervical lymph node resection. Neck MRI showed that the epicenter of the left lobe mass was in the thyroid gland and that it did not originate from the surrounding thyroid cartilage (Fig. 1b).

### Pathological findings

Macroscopic examination of the resected specimen revealed a lobulated mass with calcification that occupied the majority of the left thyroid lobe (Fig. 1c) and two homogeneous, oval nodules in the right thyroid lobe (Fig. 1d).

**Fig. 2:**
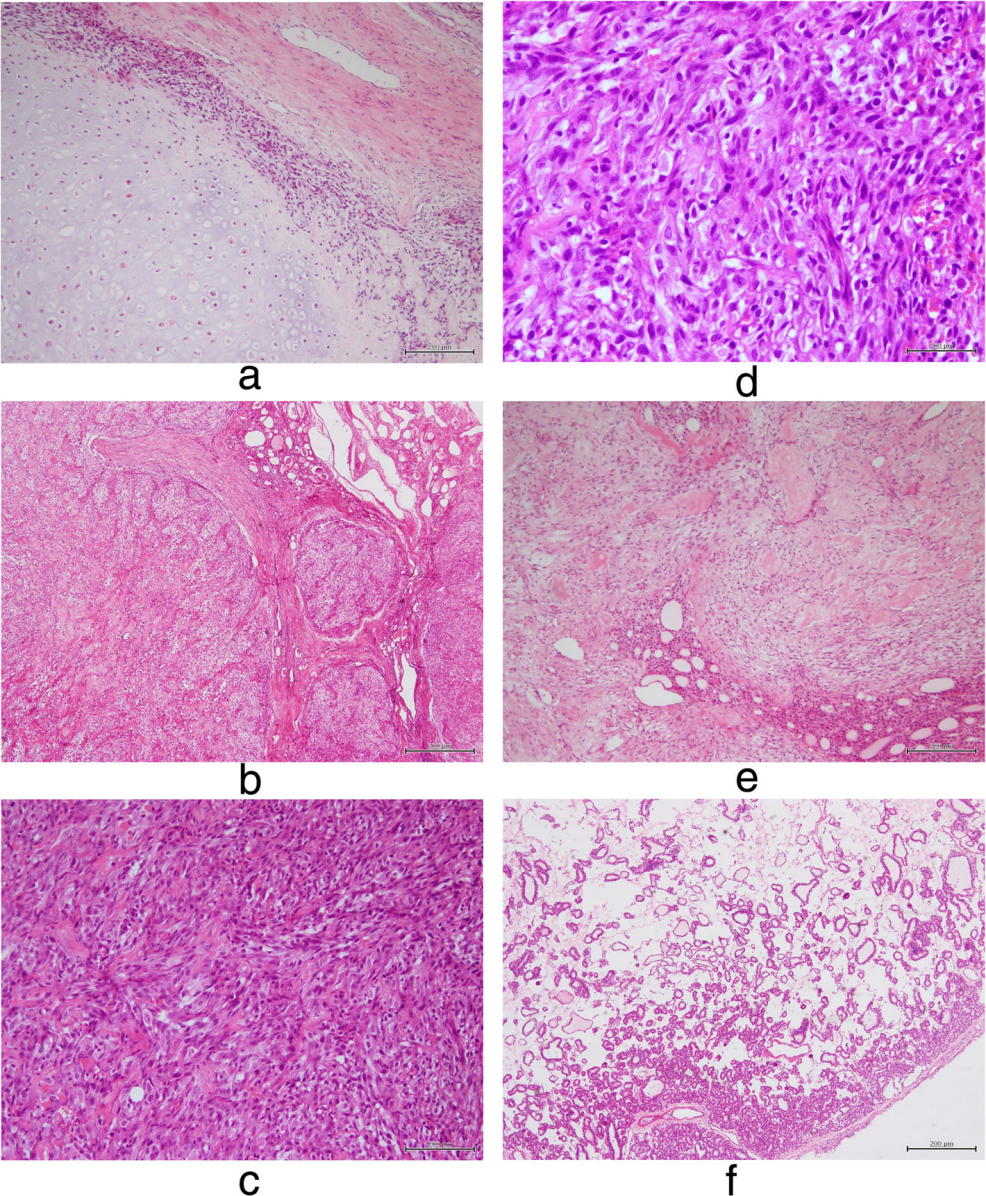
**a**. Pathological section (hematoxylin and eosin [HE] staining, × 100) showing an area with features of low-grade chondrosarcoma. **b**. The tumor in the left thyroid lobe infiltrating the remaining thyroid tissue (HE, × 40). **c**. A high-grade spindle cell sarcoma can be seen in some areas (HE, × 200). **d**. The spindle cells exhibit extensive pleomorphism, atypia, and a high mitotic rate (HE, × 400). **e**. The gray-white nodule in the right thyroid lobe comprises a prominently collagenous stroma and some bland spindle cells, which are infiltrating the surrounding thyroid tissue (HE, × 100). **f**. The brown nodule in the right thyroid lobe is a follicular adenoma (HE, × 40)

Histopathologically, the majority of the tumor in the left thyroid lobe comprised low-grade hyaline-type cartilage, and some spindle cells were observed around the hyaline cartilage (Fig. 2a). In some areas, the tumor infiltrated the remaining thyroid tissue (Fig. 2b). A high-grade spindle cell sarcoma could be seen in some areas (Fig. 2c), in which the high-grade sarcoma cells exhibited extensive pleomorphism, atypia, and a high mitotic rate (Fig. 2d). One of the two nodules in the right thyroid lobe had a gray-white appearance, and it comprised a prominently collagenous stroma and some bland spindle cells infiltrating the surrounding thyroid tissue (Fig. 2e). The other nodule had a brown appearance; it was completely enclosed within a thin, fibrous capsule and exhibited normofollicular and microfollicular architecture (Fig. 2f). We carefully examined all hematoxylin and eosin–stained sections; however, we did not observe any definite necrosis.

### Immunohistochemical findings

**Fig. 3:**
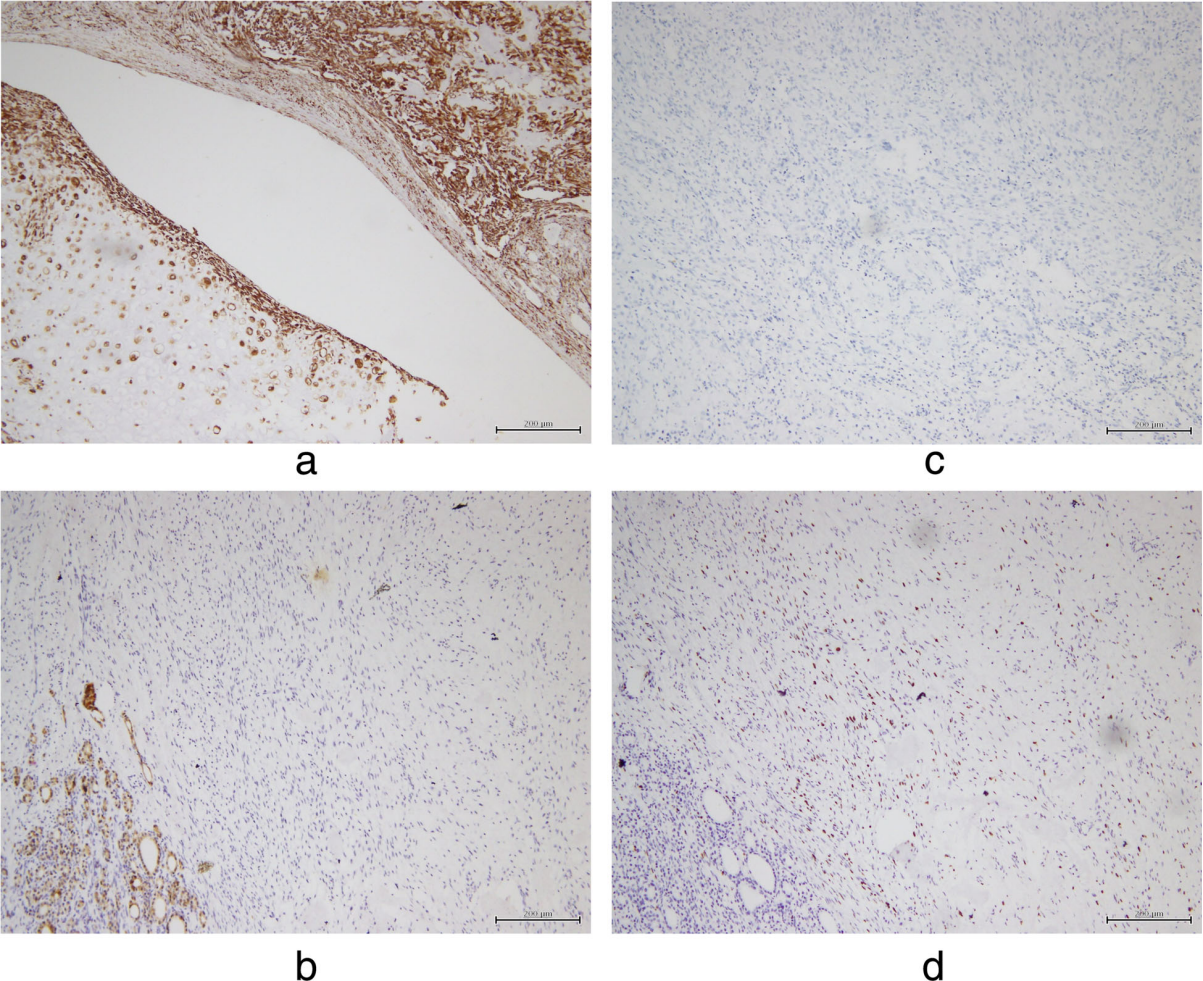
Immunohistochemical staining of the specimens. **a**. The low-grade hyaline-type cartilage and the spindle cells in the left thyroid specimen are positive for vimentin (90%) (× 100). **b**. The spindle tumor cells in the resected right thyroid lobe are negative for cytokeratin (× 100). **c**. The spindle tumor cells in the resected left thyroid lobe are negative for cytokeratin (× 100). **d**. The spindle cells in the resected right thyroid lobe show high expression of Ki-67 (20%) (× 100)

Table 1 shows the summary of immunohistochemical findings. The spindle tumor cells in both thyroid lobes were positive for vimentin (Fig. 3a) but negative for cytokeratin (Fig. 3b and c) and epithelial membrane antigen. These spindle cells showed high expression of Ki-67 (Fig. 3d). The low-grade hyaline-type cartilage in the left thyroid tumor was positive for vimentin (Fig. 3a). The positive rate of expression of vimentin in both the thyroid tumors was 90%. The positive rate of expression of Ki-67 in the spindle cells of the left thyroid tumor was 50%. The positive rate of expression of Ki-67 in the spindle cells of the right thyroid gray-white nodule was 20%.

**Table 1:** Immunohistochemical profiles of the thyroid tumor components

Marker	Spindle cells of the left thyroid tumor	Low-grade hyaline-type cartilage in the left thyroid tumor	Spindle cells of the gray-white nodule in the right thyroid tumor
Vimentin	+ 90%	+ 90%	+ 90%
Cytokeratin	–	–	–
EMA	–	–	–
Ki-67	+ 50%	/	+ 20%
Calcitonin	–	/	–
Thyroglobulin	–	/	–
Desmin	–	/	–
SMA	–	/	–
CD34	–	/	–
S100	–	/	–
PAX8	–	/	–

*EMA* epithelial membrane antigen, *PAX8* paired box-8, *S100* soluble protein 100, *SMA* smooth muscle actin

The patient received postoperative radiotherapy and was free of local and regional recurrence or distant metastases at the 8-month follow-up evaluation.

## Discussion and conclusions

To our knowledge, this is the first reported case of ATC with a chondrosarcomatous component, an extremely rare variant [4]. The highly variable microscopic appearance of ATC can be broadly categorized into three patterns that can occur alone or in any combination: sarcomatoid, giant cell, and epithelial. The secondary features of ATC include acute inflammation, macrophage infiltration, and osteoclast-like multinucleated giant cells. The other reported rare variants of ATC include paucicellular variant [2], angiomatoid [3], lymphoepithelioma-like, and small-cell variant.

The tumor boundary was clear in the left thyroid lobe. In some areas, mitotically active spindle sarcoma-like tumor cells were observed. Immunohistochemical studies revealed that these spindle cells were positive for vimentin but negative for cytokeratin, paired box-8, epithelial membrane antigen, calcitonin, and thyroglobulin; furthermore, Ki-67 expression was high (50%) in these cells. In other areas, there was abundant cartilage matrix production and irregularly shaped lobules of cartilage, sometimes separated by fibrous bands, were present. The chondrocytes were mildly/moderately atypical with enlarged, hyperchromatic nucleoli. Importantly, dedifferentiated chondrosarcoma has a similar histological appearance. Interestingly, in the right thyroid lobe, the gray-white nodule had a clear boundary, and some bland spindle cells were observed in the prominently collagenous stroma. Furthermore, 20% of the cells showed Ki-67 expression; these spindle cells infiltrated the adjacent thyroid tissue.

Several studies have reported that ATC exhibits a variable immunophenotype. Immunoreactivity for cytokeratin is present in 40–100% of ATC cases depending on the series, and vimentin is consistently present in the spindle cell component [9–11]. Sarcomatoid ATC closely resembles a large variety of soft tissue sarcomas. When a well-differentiated component is lacking and immunohistochemistry fails to demonstrate an epithelial differentiation, similar to that observed in the present case, definitive diagnosis can be challenging [12]. However, it should be noted that primary sarcomas of the thyroid are very rare, and one study has suggested that all sarcomatoid tumors of the thyroid gland should be regarded as ATCs [12]. In the present case, the patient had no history of chondrosarcoma, and he was clinically evaluated to exclude metastases; therefore, a diagnosis of ATC with chondrosarcomatous differentiation was made.

The median survival time of patients with ATC following diagnosis is approximately 5 months [13]. A study including nine patients with dedifferentiated chondrosarcoma reported that all nine patients died of lung metastases, with a median survival time of 10 (range, 3.4–18.8) months [14]. Therefore, the prognosis is dismal in both tumor types. The cause of death is attributable to upper airway obstruction and suffocation, which often develop despite tracheostomy, in 50% of patients with ATC; in the remaining patients, the cause of death includes complications of local and distant disease or therapy [15]. If the tumor appears resectable, an attempt should be made for total thyroidectomy with complete gross tumor resection together with selective resection of all involved local or regional structures and lymph nodes [1]. The most effective treatment modality for chondrosarcoma is surgery with wide *en bloc* resection to obtain an adequate histologically clear margin; the surgical approach depends on the histological grade, tumor extension, and location. Radiotherapy and chemotherapy do not appear to have a significant effect on survival and should be used for palliative purposes [16, 17]. The National Comprehensive Cancer Network guidelines suggest that the treatment for dedifferentiated chondrosarcoma should be similar to that for osteosarcoma. The current management strategy for newly diagnosed osteosarcoma includes neoadjuvant chemotherapy, followed by the surgical removal of the primary tumor and all clinically evident metastatic disease, with the addition of adjuvant chemotherapy following surgery [18]. However, in addition to the lack of convincing evidence regarding the benefits of chemotherapy, the associated toxicity in older patients with dedifferentiated chondrosarcoma generally rules out this modality as a standard treatment [14]. However, ATC is histologically distinct from differentiated thyroid cancer; furthermore, due to its highly aggressive nature, aggressive postoperative radiotherapy and chemotherapy are typically recommended. A study conducted at the Zhejiang Cancer Hospital investigated prognostic factors in 56 patients with ATC and reported that the median survival time was 4.5 months and overall 1-year survival rate was 5.4%. Furthermore, the overall 1-year survival rate of 12.5% in patients who were treated with surgery in combination with radiotherapy was higher than that in patients who received other treatments [19]. Therefore, our patient underwent total thyroidectomy with cervical lymph node resection and received postoperative radiotherapy. The patient was free of local and regional recurrence or distant metastases at the 8-month follow-up evaluation, which we speculate was due to the relatively clear tumor boundaries that facilitated a complete resection.

This first report of a patient with an unusual ATC containing a chondrosarcomatous component further expands the histopathological spectrum of ATC, which includes dedifferentiated chondrosarcoma as the main differential diagnosis. The prognosis is poor for both ATC and dedifferentiated chondrosarcoma, and complete tumor resection remains the primary treatment. Long-term observation is necessary to better elucidate the biological behavior of ATC containing a chondrosarcomatous component.

## Data Availability

The datasets used and/or analyzed during the current study are available from the corresponding author upon reasonable request.

## Abbreviation

ATC: Anaplastic thyroid carcinoma
